# The Significance of Biobanking in the Sustainability of Biomedical Research: A Review

**DOI:** 10.29252/ibj.24.4.206

**Published:** 2020-02-12

**Authors:** Mahshid Zohouri, Abbas Ghaderi

**Affiliations:** Shiraz Institute for Cancer Research, School of medicine, Shiraz University of Medical Sciences, Shiraz, Iran

**Keywords:** Biobank, Cancer, Iran, Personalized medicine

## Abstract

Biobank, defined as a functional unit for facilitating and improving research by storing biospecimen and their accompanying data, is a key resource for advancement in life science. The history of biobanking goes back to the time of archiving pathology samples. Nowadays, biobanks have considerably improved and are classified into two categories: diseased-oriented and population-based biobanks. UK biobank as a population-based biobank with about half a million samples, Biobank Graz as one of the largest biobanks in terms of sample size, and IARC biobank as a specialized WHO cancer agency are few examples of successful biobanks worldwide. The present review provides a history of biobanking, and after presenting different biobanks, we discuss in detail the challenges in the field of biobanking and its future, as well. In the end, ICR biobank, as the first cancer biobank in Iran established in 1998, is thoroughly described.

## Introduction

**Definition of biobank**

The term “biobank” refers to the collection of plants and animals as well as human specimens^[^^1^^]^. Herein, we talk about human biobanks as a connecting unit between clinic and research, the development of which is necessary to enable valuable translational research^[^^2^^]^. Biobank is globally defined as a functional unit to facilitate the access of researchers to high-quality samples and their connected data^[^^3^^]^. BBMRI has also described biobanking as a unit containing biological samples and associated information, which is crucial raw materials for the development of biotechnology, human health, and research in life science^[^^4^^]^. Generally, the definition of biobank can be divided into three parts: biological human materials, connected information, and legal issues such as consent and individual’s/patient’s data protection and safety^[^^5^^]^.

**Importance of biobanking**

As reported by TIME magazine in 2009, biobank was among the list of the “10 ideas changing the world right now”, which asserts the importance of biobanking^[^^6^^]^. Biomedical research is greatly dependent on high-quality specimens and their large numbers. The limited number of biospecimen samples will restrict the scope of questions that may be addressed in the field of research, hence preventing researchers from answering larger questions and generalizing findings across patient populations. On the other hand, the quality of biospecimens affects the quality of biomedical research so that biospecimens of unknown or poor quality can invalidate research findings^[^^7^^,^^8^^]^. Meanwhile, progress in the fight against cancer is influenced by the limited access to high-quality biospecimens^[^^9^^]^. Translational research, defined as an enterprise of gathering basic sciences to manufacture new treatment options for patients, is highly dependent on the size of the resources^[^^10^^,^^11^^]^. Nowadays, translational “cancer” research is thriving to reverse the rising gradient of cancer mortality and morbidity by using molecular and clinical data from each patient, to develop more targeted therapies with lower adverse effects, as well as to determine disease predisposition that could help early detection and prevention of cancer^[^^9^^]^.

**History of biobanking**

Biobanking is a “long-existing” activity and at the same time a progressively complex “young discipline”. The history of biobanking can stretch as far as the time when clinical samples of pathology were first archived^[^^2^^]^. However, the history of real biobanks is not long and dates back to about 30 years ago when the first repositories randomly collected information and samples created^[^^5^^]^. For over 100 years, human specimens have been stored at worldwide institutions ^[^^12^^]^. Mayo Clinic, one of the oldest medical centers, has archived all tissue slides and blocks since 1907. HeLa cell is the oldest (February 1951) and most commonly used human cell line from cervical cancer^[^^13^^,^^14^^]^. It is a novel example of this kind of collection that has unimaginably influenced research. Development of polio virus vaccine, success in gene mapping, cancer research, the effects of radiation and toxic substances, research in AIDS and many other fields of medicine are owed to this special biospecimen^[^^15^^]^. Moreover, HeLa cells have been used to test human sensitivity to glue, cosmetics, tape, and many other products^[^^16^^]^. Biobanking has been changing since the past 30 years, starting from the small, university-based repositories and reaching the current large, population-based biobanks. Similarly, the biospecimens-associated data have become more complex, going from basics (i.e. data collection) to modern-day extensive information sets (i.e. participant phenotype, genetics, proteomics, etc.)^[^^1^^]^. 

**Category of biobanks**

The classification of biobanks is mainly performed based on multiple designed approaches. Rebulla *et al.*^[^^17^^]^ have categorized biobanks into six categories, including tissue (collection during diagnostic procedures for clinical pathology), twin, population, disease, organ, and nonhuman biobanks. One of the most broadly accepted classifications is conducted by BBMRI, which considers two types of biobanks: population-based biobanks and disease-oriented biobanks^[^^5^^,^^18^^]^.

***Population-based biobanks***

Longitudinal population-based biobank is the most common format. In such a population cohort, at the time of entry and at certain times in follow-up, blood or isolated DNA, along with data about lifestyle, family history, environmental exposure, etc., is collected from a general population who might or might not have a specific disease. Its main goal is achieving susceptibility biomarkers, environmental risk factors, and predisposing genetic variants in healthy individuals. A major issue arises from this category with this format is that investigation can only be initiated after follow-ups for about 10–15 years when they have access to the minimum amount of samples of a specific disease^[^^10^^,^^18^^,^^19^^]^.

***Disease-oriented general biobanks***

This type of biobanks (i.e. tumor banks) focuses on discovering biomarkers of disease as well as predicting progression of the disease, mortality, and response to treatment, through prospective and/or retrospective collections of samples (tissue and isolated cells) and blood derivate (proteins/RNA/DNA) or other body fluids. Samples are usually associated with clinical data, the amount of which will determine the biological value of the sample^[^^10^^,^^18^^]^. Disease-oriented biobank provides an infrastructure for the collection and storage of samples from different types of diseases; therefore, the high number of represented diseases is their specific strength. A large number of samples lead to the identification of different pathways, which in turn leads to new target therapies that are key to the advancement of personalized medicine^[^^20^^]^. The two mentioned types of biobanks are connected as a result of prospective population-based biobanks complementing disease-oriented biobanks, in which they can find predictive biomarkers for the onset of diseases. An optimal scenario for the case-control studies would happen if we follow individuals in years and use their samples for both case and control after developing the desired disease^[^^4^^]^. 

**Cancer biobanks**

Cancer biobanks, as a subtype of disease-based biobanks, are essential for evolution in cancer treatment. Almost all aspects of cancers are affected by biobanking like pathogenesis, diagnosis, treatment, and prognosis^[^^21^^]^. The oncology of the 20^th^ century focuses on generic therapeutic regimens based on phenotypic and morphologic tumor classification. Unfortunately, such therapy was not effective for all tumors with the same morphology or phenotype and also had unpredictable adverse effects. In contrast, today’s oncology gives more attention to early detection and prevention, molecular classification of tumors, characterization oncogenesis pathways, response prediction by pharmacogenomics, and targeted therapies as a part of personalized medicine^[^^2^^,^^22^^]^. The rapid progress of molecular pathology, genetic epidemiology, and pharmacogenomics in different types of cancer is owed to advances in biobanking^[^^23^^]^. Due to the necessity of research in the field of cancer, several cancer biobanks have been established. The IARC, as the specialized cancer agency of the WHO, was established in May 1965 to promote cancer research through international collaboration. Today, IBB has nearly six million samples from 600,000 subjects, which help both population-based and disease-based collections^[^^24^^]^. Also, BC cancer center, as the first cancer treatment center in Vancouver (established in 1938), has now about 90,000 donors. Under the supervision of BC cancer center, Personal Response Determinants in Cancer Therapy program started as a pilot project in 2006 in Vancouver with the registration of 10,000 participants till 2014^[^^25^^,^^26^^]^. 

**Largest biobanks**

In May 2018, biobanking.com introduced the 10 largest biobanks in the world^[^^24^^]^. UK biobank with the coordination of 500,000 volunteers since 2006^[^^27^^]^, China Kadoorie biobank by enrollment of 512,891 individual since 2004^[^^28^^]^, BBJ Project with 200,000 participants since 2003^[^^29^^]^, and Biobank Graz with an automated systemic collection of 7.5 million samples for 30 years are among the largest biobanks^[^^30^^]^. By data collection in cohort studies of biobanks, about 79,000 cancer diagnoses and 825,927 cases of single-nucleotide polymorphism have been recorded by UK biobank and also 46 new associations for biochemical and hematological traits were reported in Japanese population by BBJ^[^^31^^,^^32^^]^. Owing to the rapid growth of biobanks and the lack of standardized and high-quality biospecimens, in late 2009, a biobank, namely caHUB, was established. Under the supervision of the US National Cancer Institute by creating evidence-based Standard Operating Procedures, caHUB is aiming to improve the field of biobanking^[^^1^^,^^33^^]^.

**Challenges in Biobanking**

The biobanking community, since its inception in early 2000, has had to overcome several challenges, including harmonized procedures, appropriate design, and sustainability, all in the framework of their legal, social, and ethical values^[^^10^^,^^34^^]^. In response to biobanking challenges, Chalmers *et al.*^[^^34^^]^ have introduced four waves, which, in the following, we introduce the main concepts based on the original references used by Chalmers *et al.*^[^^34^^]^ and clarify them based on the literature review.

***“Establishing biobank governance and management frameworks” ***

Large tissue biobanks heralded concerns about governance and security issues like consent and privacy. Due to the development of biobanks for long-term research, the previous approach of “one study/one informed consent” has faced challenges since, at the time of sample collection, cases for future research are unknown. On the other hand, it is impractical to obtain consent from each participant for each research project, which results in the growing acceptance of broad consent for taking samples and information and storing them^[^^34^^,^^35^^]^. Another problem in this area is the challenging ethical issues related to using an individual’s linked data, genetic information, etc. while considering the participants’ confidentiality and privacy^[^^36^^]^. With respect to this problem, OECD released information privacy principles in 1980 to create a reference for consistency. Therefore, policy makers understand that biobanking needs specific governance frameworks. The OECD published guidelines on human biobanks and genetic research databases in 2009. However, the variability of biobanks in scale, size, participant’s health status, the scope of research, etc., has presented challenges for consistent regulatory responses^[^^37^^]^.

***“Collaboration and standardization”***

In a non-collaborative state, access to biospecimens is isolated to an investigator’s own institution, and research questions are limited by the scope of the available samples. As a result, collaboration at national or international level is essential. Poor or unknown quality of biospecimens can lead to doubts regarding research findings. The disparity in research findings arises from the lack of standardized procedures for collecting, storing, processing, and annotating biospecimens^[^^7^^]^. One evidence is a report by Moore *et al.*^[^^9^^]^ who showed that only 30 out of 660 commercial tests available for measuring gene alterations, and germline mutations can be used to predict response to a specific treatment. It is also apparent that standardization of practices, procedures, and policies is key to achieve optimal results. To overcome the aforementioned challenges, the International Society of Biological Environmental Repositories was established to take a leading international role in standardizing the preservation and storage of biobanked material. Also, BRN under the supervision of OBBR was established in 2005 to coordinate and support systematic investigation with regards to collection, processing, and storage of human biospecimens. However, standardization processes might be a threat to smaller biobanks, because they might not be able to meet the benchmarks^[^^33^^,^^34^^]^.

***“Seeking sustainability amidst ongoing challenges”***

Sustainability is one of the most troublesome issues in the field of biobanking. The financial value, operational efficiency, and social acceptability are some of the metrics in measuring the sustainability of biobanks^[^^38^^]^. As asserted by Professor Hank Greely, biobanking is “staggeringly expensive”^[^^39^^]^, which emphasizes the necessity of developing and maintaining sustainable business practices. The National Biobanking Strategy Committee suggested in their 2013 meeting that without a more stable core-funding stream, the future of biobanks, especially cancer ones, is in doubt. Cost-recovery is one of the suggested solutions to this issue; however, the model’s success depends on the number of outgoing samples, providing catalogs, and advertising for marketing research data^[^^34^^]^. Nowadays, it has become obvious that the success of biobanks is linked to establishing and retaining public trust; failure of the Icelandic Health Sector Database, as one of the earliest population biobanks, happened because of lacking this issue^[^^40^^]^. It has also been predicted that the highly-expensive population biobank would not survive for much longer^[^^34^^]^. Therefore, biobanks and society are inseparable. It means that society needs biobanks to support its health, while society can help biobanks by providing financial resources, generalized support, and cooperation and trust of participants to guarantee its sustainability^[^^41^^]^.

***“Biobanks future and new biobank models”***

The last wave concerns the future of biobanks. So far, as best described by Turner *et al.*^[^^42^^]^, we can conclude that “Biobanks are caught directly between the values and rights of the participants and the potential commercial and scientific value of the samples and data, and at the same time, have to construct a business model that will ensure the long-term sustainability of the biobank”; therefore, to date, there is no standardized, generally accepted biobank model.

**Future of biobanks**

***Networking and , national and international biobanks***

Until now, biobanking has enabled research studies and helped progress in the understanding of disease pathogenesis. However, the need for biobank networking to assist in novel discoveries is still unsatisfied. The idea of national and international biobank networking is one of the newest areas in this field and predicted to have an important effect on prevention and treatment of diseases, especially cancer. Such networking enables the investigation of rarer diseases, validating molecular signatures that have multiple parameters, helping pharmaceutical companies to work on data from different ethnicities and also discussing the role of environmental risk factors while focusing on genetic background^[^^4^^,^^43^^]^. Key publications related to biobank networking are the Cancer Incidence in Five Continents series and GLOBOCAN, which are coordinated by IARC^[^^44^^]^. At the national level, networks of biobanks began to emerge and proliferate during the 1990s. Up until now, countries with the most large-scale biobanks are the UK with 15 and the USA with 14 corporators^[^^45^^]^. Central Research Infrastructure for molecular Pathology located at the Institute for Biomedical Engineering in Germany is another example of transnational networking between different European tissue banks. Their archives contain about five million Formalin-Fixed Paraffin-Embedded and 50,000 frozen tissue samples^[^^4^^,^^46^^]^. 

***Living biobanks***

Recently, in the field of chronic disease treatment, especially cancer, the inefficiency of the “one-size-fits-all” approach has been proven. Today, with the development of human genetics, pharmacogenomics, and the success of human gene mapping, the “one dose-one patient” approach in the growing field of personalized medicine needs to be replaced. In this field, the major challenge is finding a link between functional genomics and pathological data while focusing on the patient’s outcome. Many different personalized tumor models have been proposed to address this challenge. One of the newest models in a three-dimensional culture tumor model named organoid^[^^47^^]^. With the improvements in this field, many tumor organoids derived from tumor specimens from the intestine, stomach, liver, mammary glands, retina, brain*, *etc.has been developed, which emphasizes the need for living biobanks^[^^47^^,^^48^^]^. Sachs *et al.*^[^^49^^]^ have recently built a living biobank with more than 100 primary and metastatic breast cancer organoid lines.

***Walking biobank and hybrid model***

Technology can contribute to research capabilities as well as ethical and legal issues surrounding it. Recently, a new form of consent called “dynamic consent” has been introduced. Dynamic consent is a personalized, digital communication purposed to connect researchers and participants. As a two-way communication, it helps participants to manage their own consent preferences over time, follow their information and samples, and at the same time, benefits researchers by enabling more efficient participants to recontact^[^^50^^]^. Based on this strategy, a new concept for biobanking has been introduced by Chalmers *et al.*^[^^34^^]^, which resembles routine blood sampling for clinical purposes. In this model, rather than spending the limited funds on the infrastructure, for any specific research question, researchers may ask the participants to “walk-in” and donate tissue or information as needed. Besides the associated sustainability, efficiency, and cost issues, this new model may seem more challenging if one person has to be called in several times, while one key benefit of the old model is that only a one-time participation is required per person. To overcome these limitations, a type of “hybrid model” has been proposed. The presented model has the ability to maintain regular tissue collection, and at the same time, long-term relationships with participants. 

***Virtual biobank***

A virtual biobank is an electronic database for biological specimens and their attached information, regardless of where the specimens are stored^[^^1^^]^. This model still depends on the traditional biobanks as a source of its data, and therefore, most of the limitations associated with the traditional ones still apply to virtual biobank. BRN and Biospecimen Resource Database, created by OBBR, are examples of virtual biobanks in the USA^[^^42^^]^. 

**Shiraz ICR, the first Iranian cancer biobank**

Developing countries, due to their high disease burden, have a large target sample population, and Iran is no exception. Cancer is the third leading cause of death among the Iranian population, which makes research in this field and the establishment of cancer biobanks are more crucial^[^^51^^]^. Nowadays, in spite of many different types of biobanks in Iran, no data has been documented on the history of biobanking or the number of biobanks. Based on our data, ICR biobank, built in 1998 at Shiraz University of Medical Sciences, is one the first in the country. Concurrent with the establishment of the Institute, its biobank was also started. Sample collection and data storage systems have recently changed a lot over time, going from “hypothesis-tailored”^[^^52^^]^ collection and manual data storage at the beginning to “hypothesis-free”^[^^52^^]^ collection with computer data storage in recent years. This attempt has brought the center’s practices closer to universal standards. 

ICR biobank, located in the city of Shiraz, has connections with several Shiraz University Hospitals for providing blood samples. At the time of a patient’s admission to a cooperating hospital, the sample and its

associated data are collected and sent to ICR with a mean delay of 1.5 hours. Subsequently, DNA is extracted and stored in special refrigerators, alongside with serum and plasma samples. Samples will then be used based on the demand of researchers after signing an agreement by the directors. Since the day of establishment, the bank has registered over 12,000 patients with samples of DNA, serum, and plasma from different types of cancers and their accompanying data (Table 1). ICR biobank has also a collaboration with other universities at national and international levels in providing requested samples or performing joint research projects. Examples of such universities include Tehran University of Medical Science and multiple universities in other provinces of the country, alongside with universities around the world such as the University of California, deCODE genetics center in Iceland, Radboud University Nijmegen Medical Center in the Netherlands, Stanford and UCLA in the USA, etc. Up to now, over 380 PubMed articles and 300 theses have been published based on our biobank resources. Publications until 2018 are available online at http://icr.sums.ac.ir/en/publications/articles.html.

**Table 1:** ICR biobank samples

**Type of cancer**	**No. of specimen**
Breast	4900
Head and neck	1160
CNS tumor	660
Bladder	650
Colorectal	492
Lung cancer	383
Non-melanoma skin cancer	242
Prostate	160
Gasteric	152
Renal	144
Bone	135
Ovary	134
Acute leukemia	120
Cervix	105
Pancreas	90
Melanoma	50
Lymphoma	40
Others	3800

**Fig. 1:**
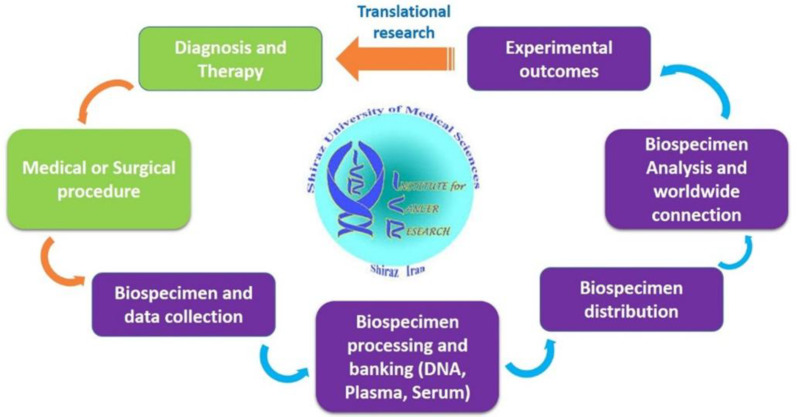
ICR biobank networking, including cancer diagnosis, medical procedure, specimen and data transfer, processing, storage, sharing, collaboration, and outcome

Overall, ICR like other biobanks in developing countries lacks the infrastructure required for standard biobanking. Moreover, because of “pick and choose”^[^^53^^]^ policy for sample collection due to limited budget, our biobank lacks different types of samples such as hair, cerebrospinal fluid, tissue, etc. Therefore, there is still a long way to reach international standards, especially with respect to informed consent, standardization of sample collection and storage, networking and most importantly, seeking sustainability in the “staggeringly expensive” area of biobanking. However, at its own pace, it has been effective for Iranian population’s cancer research. (Fig. 1 summarizes the network of ICR biobank). More data is available online at http://icr.sums.ac.ir. 
